# Notch signals in the endothelium and cancer "stem-like" cells: opportunities for cancer therapy

**DOI:** 10.1186/2045-824X-4-7

**Published:** 2012-04-09

**Authors:** Jian-Wei Gu, Paola Rizzo, Antonio Pannuti, Todd Golde, Barbara Osborne, Lucio Miele

**Affiliations:** 1 University of Mississippi Cancer Institute, Jackson, MS, USA; 2 Cardiovascular Research Center "Salvatore Maugeri" Foundation, Lumezzane, Italy; 3 Department of Neurobiology, University of Florida, Gainesville, FL, USA; 4 Department of Veterinary and Animal Sciences, University of Massachusetts at Amherst, Amherst, USA; 5 Ergon Professor of Medicine and Pharmacology, University of Mississippi Medical Center, 2500 N. State St., Suite G751-5, Jackson, MS 39216, USA

## Abstract

Anti-angiogenesis agents and the identification of cancer stem-like cells (CSC) are opening new avenues for targeted cancer therapy. Recent evidence indicates that angiogenesis regulatory pathways and developmental pathways that control CSC fate are intimately connected, and that endothelial cells are a key component of the CSC niche. Numerous anti-angiogenic therapies developed so far target the VEGF pathway. However, VEGF-targeted therapy is hindered by clinical resistance and side effects, and new approaches are needed. One such approach may be direct targeting of tumor endothelial cell fate determination. Interfering with tumor endothelial cells growth and survival could inhibit not only angiogenesis but also the self-replication of CSC, which relies on signals from surrounding endothelial cells in the tumor microenvironment. The Notch pathway is central to controlling cell fate both during angiogenesis and in CSC from several tumors. A number of investigational Notch inhibitors are being developed. Understanding how Notch interacts with other factors that control endothelial cell functions and angiogenesis in cancers could pave the way to innovative therapeutic strategies that simultaneously target angiogenesis and CSC.

## Introduction

The endothelium is a key regulator of vascular integrity and function. Endothelial cell functions and gene expression profiles are controlled by cytokines, hormones and metabolic products, as well as by mechanical stimuli such as shear stress caused by changes in blood flow [1]. Endothelial cells play a major role in the creation of supplemental blood vessels in ischemic tissues following vascular obstruction. This process is "hijacked" by cancer, which depends on neo-angiogenesis and vasculogenesis for growth and invasion. Endothelial cells are also an important component of the "vascular niche" for cancer stem-like cells (CSC) [2]. A number of pathways, including vascular endothelial growth factor (VEGF) and its receptors (VEGFRs), basic fibroblast growth factor (bFGF), transforming growth factor beta (TGFβ), and platelet-derived growth factor (PDGF) with their receptors, angiopoietin/Tie and ephrin/Eph, regulate vasculogenesis and angiogenesis [3]. Notch signaling, directly or by cross-talking with other pathways, plays a major role in modulating endothelial cells functions [4]. Additionally, Notch signaling has emerged as one of the master pathways in CSC [5]. This review summarizes the current data on the effects of Notch signaling in endothelial cells and CSC and how this modulation can be exploited for therapeutic purposes.

## The Notch pathway

**Figure 1:**
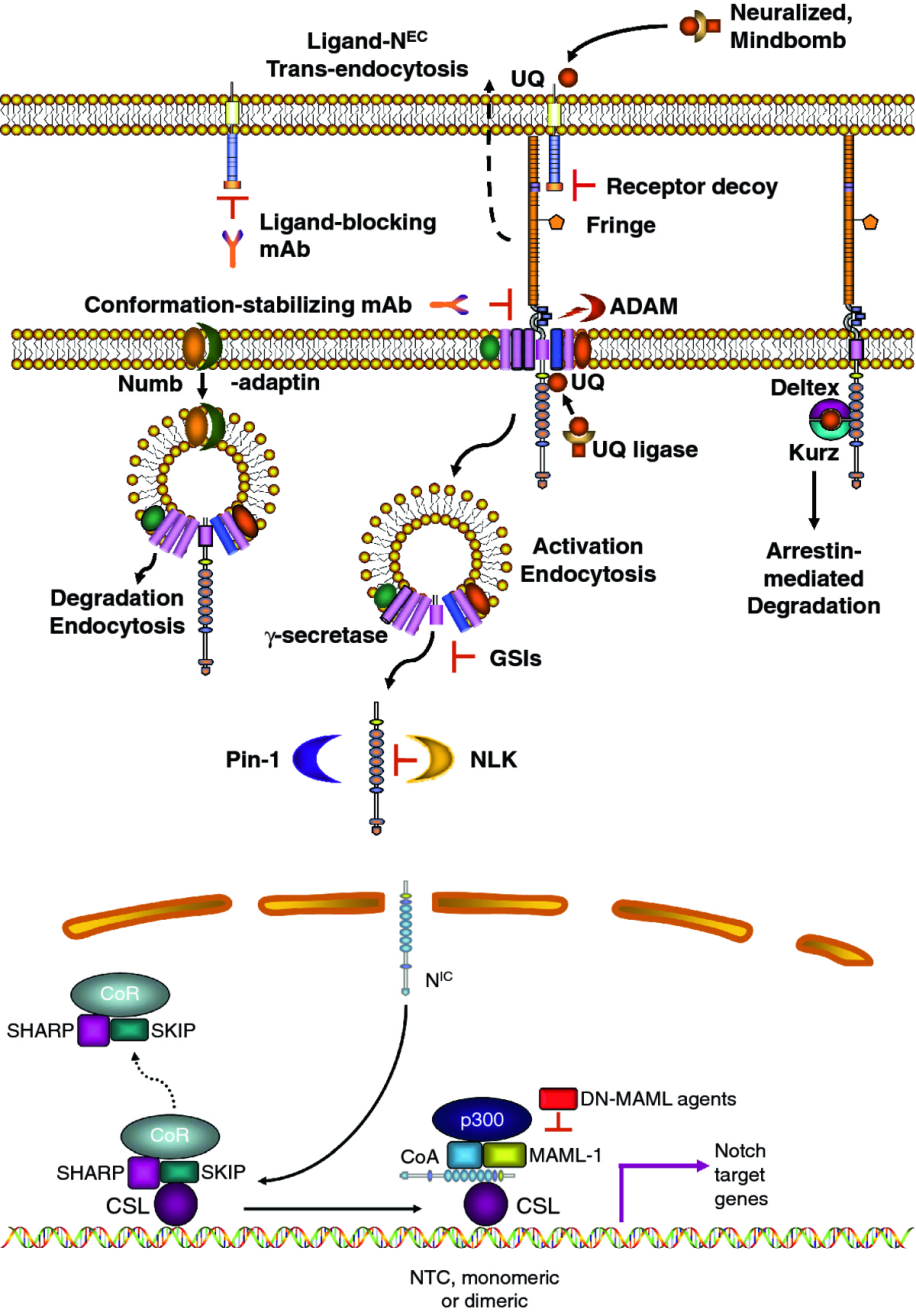
**A simplified diagram of canonical Notch signaling: A: membrane and cytoplasmic events**. In ligand expressing cells, ligands are ubiquitinated (UQ) by E3 ligases Mindbomb and Neuralized, endocytosed and "activated". "Active" ligands bind Notch receptors, dissociating N^EC ^from N™. The complex ligand- N^EC ^is trans-endocytosed into the ligand-expressing cell, perhaps providing mechanical energy to separate N^EC ^from N™. Some ligands expressed in cis can bind Notch on the same cell, causing cis-inhibition. Ligand-induced N^EC ^separation unmasks the ADAM cleavage site (red), which is cleaved by ADAM10 or ADAM17, producing N^EXT ^and a short peptide which is released. N^EXT ^is cleaved by γ-secretase, at the membrane or during endocytosis, generating N^IC^. This process is facilitated by adaptor-associated kinase AAK1 [101] and may require mono-ubiquitination. The release of N^IC ^from endosomes (or the selection of cleavage site by γ-secretase) may require endosome acidification (H^+^) by aquaporin Bib. The stability of N^IC ^is regulated by factors such as Pin-1 prolyl isomerase and NLK kinase. Endocytosis can lead to ligand-independent Notch activation catalyzed by γ-secretase. In the absence of non-visual β-arrestin Kurz, Deltex may lead to Notch endocytosis and activation. The amount of Notch available at the membrane is controlled by many endocytosis-recycling mechanisms. Several E3 ligases (Itch, CBL, Nedd4, the Deltex-Kurz complex) can target Notch for degradation. The ESCRT complex and lgd in Drosophila (and presumably their homologues in mammals) control Notch degradation, and their loss causes accumulation of Notch in endosomes and ligand-independent activation. In actively dividing cells, Numb/ACBD3 asymmetrically partitions to one daughter cell, causing selective Notch degradation in it. GSI, monoclonal antibodies (mAbs) to Notch receptors and ligands and Notch decoy molecules have been used effectively in vivo to inhibit Notch signaling. B: nuclear events. N^IC ^is transported to the nucleus, where it causes the dissociation of the co-repressor complex including SHARP, SKIP and several other proteins (CoR) from CSL. Notch, CSL and MAML form a tertiary complex which in turn recruites p300 and other coactivators (CoA) to the chromatin and forming the NTC that activates transcription. The NTC can form heterodimers on the chromatin with other NTCs or supramolecular complexes with other transcription factors, modulating the choice of genes regulated by Notch. Dominant negative (DN) MAML constructs or peptidomimetic agents have been used in vivo to inhibit Notch-mediated transcriptional activation (see reference 5 for review).

Notch signaling is a highly conserved pathway that controls cell fate decisions in metazoans from invertebrates to mammals [6,7]. It is a short range communication system between two adjacent cells, based on ligand-activated receptors. In mammals there are four paralog receptors (Notch1, -2, -3 and -4) and five canonical ligands (Delta-like or DLL1, 3, 4 and Jagged1 and 2). Both receptors and ligands are type I membrane-spanning proteins Receptors are heterodimers consisting of an extracellular subunit (N^EC^) non-covalently bound to a transmembrane subunit (N™). Both subunits derive from a single precursor that is cleaved in the trans-Golgi by a furin-like protease. Ligand binding to N^EC ^induces a conformational change that allows subunit dissociation. This is followed by the first proteolytic cut by a surface protease ADAM (A Disintegrin And Metalloprotease) which removes a short extracellular fragment of N™ and creates a membrane-tethered intermediate (Notch extracellular truncation or NEXT). NEXT is a substrate for γ-secretase, an intramembranous protease complex. γ-Secretase in turn generates the active form of Notch (Notch intracellular, N^IC^) which translocates to the nucleus where it binds transcription factor CSL (CBF-1, Suppressor of Hairless, Lag-1), also known as RPB-Jκ (recombinant signal binding protein 1 for Jκ) in mice. N^IC ^binding displaces a co-repressor complex, promotes the recruitment of co-activator molecules and the transcription of numerous Notch target genes (Figure 1). The best known Notch targets include the Hes (hairy/enhancer of split) and Hey (Hes-related proteins) families and Nrarp (Notch-regulated ankyrin repeat protein). These and other Notch targets regulate further downstream genes which can either maintain cell in an uncommitted state or induce differentiation. The mechanistic reasons for these differences remain unclear. Cyclin D1, cMyc, and many other genes that control cell proliferation, differentiation and apoptosis are also influenced by Notch [8]. Although this pathway appears deceptively simple and is theoretically identical for all 4 Notch paralogs, exceedingly complex mechanisms regulate Notch signal intensity and paralog-specific effects. These are described in our recent review [5], and summarized diagrammatically in Figure 1. In addition to embryonic development, the Notch pathway controls multiple cell fate decisions during adult life, including stem cells maintenance, differentiation and proliferation as well as apoptosis in continuously renewing tissues such as the epidermis, the intestinal epithelium and the endothelium.

## Role of Notch during embryonic vascular development

Vascular development is modulated by Notch signaling, which is active in both endothelial and smooth muscle cells. In particular, endothelial cells express Notch receptors 1, -2 and -4 and ligands Jagged1, DLL4 and DLL1 while vascular smooth muscle cells (VSC) are characterized by Notch3 expression. [9,10]. During embryonic development, Notch induces differentiation of angioblasts to endothelial cells, and controls cell fate specification of endothelial cells into arterial or venous identities [11]. Mouse embryos with Notch1 loss of function or double Notch1 and Notch4 loss of function mutations display severe defects in vascular development [12]. Endothelial-specific knockout of Jagged1 results in an embryonic lethal phenotype with absence of smooth muscle actin [13]. Loss of Notch3 produces dilated arteries with abnormal elastic laminae [14] Mice homozygous for Jagged1 loss of function mutation die from haemorrhage early during development [15]. Consistently with the major role played by Notch during vascular development, two human cardiovascular diseases are associated with genetic alterations of this pathway. Mutations of Notch3 cause CADASIL (Cerebral Autosomal-Dominant Artheriopathy with Subcortical Infarcts and Leukoencephalopathy), characterised by stroke and dementia due to vascular lesions [16]. Alagille syndrome is a pleiotropic developmental disease caused by mutations of Jagged1 and characterized by congenital heart defect with cardiovascular anomalies [17].

## Role of Notch in vascular homeostasis and function during postnatal life

### Ischemic tissues and tumor angiogenesis

Angiogenesis requires stimulation of vascular endothelial cells through the release of angiogenic factors. Of these, vascular endothelial growth factor A (VEGF-A) is the most critical regulator of vascular development [18]. VEGFR2 regulates most of the endothelial cell response to VEGF-A, including cell migration, proliferation, survival, permeability and sprouting of new blood vessels from pre-existing ones [19]. Sprouting begins with VEGF-A induction of filopodia on specialized endothelial cells, the "tip" cells, which are guided by a gradient of VEGF-A [20]. For productive angiogenesis, branching must be limited to "tip" cells and simultaneously inhibited in the adjacent cells, known as "stalk" cells, characterized by lack of protrusive activity. Endothelial cells dynamically compete for the "tip" cell position, and the selection between "tip"- and "stalk" cell fate depends on the interplay between VEGF and Notch pathways which interact at several levels to generate a highly organized blood vessel network [21]. According to a model supported by a wealth of experimental data, VEGF-A induces expression of DLL4 in endothelial "tip" cells [22], which in turn activates Notch on the adjacent endothelial cells dampening their response to VEGF-A and conferring a "stalk" phenotype [23]. Notch activation in human umbilical vein cells (HUVEC) decreases their response to VEGF-A through downregulation of VEGFR-2 (Taylor 372-383) and upregulation of VEGFR-1, a VEGFR isoform with weak tyrosine kinase activity [24-26]. VEGFR-1 regulates sprout formation also by production of sFlt-1, a soluble form of VEGFR-1 that antagonizes VEGF signaling [27,28]. Directionality of the guided sprouting process is thus achieved through a population behavior, in which the migration influenced by the VEGFR-DLL4-Notch interplay, continues toward the highest concentration of VEGF-A [29]. This phenomenon is reminiscent of classical "lateral inhibition" during Drosophila neurogenesis. Ectodermal cells differentiating towards a neuronal fate prevent adjacent cells from undergoing the same fate by expressing Notch ligand Delta and activating Notch in adjacent cells [6].

Consistently with the model described above, blockade of DLL4 with specific monoclonal antibodies in experimental tumors leads to excessive branching and unproductive angiogenesis [30]. Similarly, inhibition of DLL4 signaling by intramuscular injection of an adenovirus encoding a soluble form of DLL4 extracellular domain impairs reparative angiogenesis in a mouse model of ischemia [31].

N-acetyl-glucosaminidation of fucose residues on the extracellular subunit of Notch, catalyzed by enzymes of the Fringe family, affects differentially Notch activation induced by Jagged or Delta-family ligands [32]. In particular, Fringe glycosylation, even though it does not reduce Jagged1 binding to Notch1, potentiates DLL1 over Jagged1 signaling, probably by a more effective promotion of Notch proteolysis following ligand binding [33]. Benedito et al. have shown that in presence of glycosylated Notch, high levels of Jagged1 in endothelial cells inhibit DLL4 signaling, leading to enhanced sprouting and promotion of angiogenesis [34]. Tumor necrosis factor α (TNFα), a cytokine abundant in many solid tumors, induces Jagged1 in endothelial cells, conferring a "tip" cell phenotype highly enriched in Jagged1, but not DLL4 [35]. Taken together, these findings indicate that the effects of Notch signaling on angiogenesis are also controlled by the relative expression levels of DLL4 and Jagged1 ligands, and by the relative affinity of Notch receptors for these classes of ligands, which in turn is dependent on Fringe-catalyzed Notch modifications. Factors that selectively control the expression of the two ligands DLL4 or Jagged1, or modulate the affinity of receptors for these ligands, could have a profound influence on tumor angiogenesis.

Lymphangiogenesis may be as important to tumor biology as hemangiogenesis, particularly for tumors that predominantly metastasize to regional lymph nodes. VEGFR-3 is expressed on lymphatic endothelium and with its ligand VEGF-C, stimulates the growth of lymphatic vessels, regulating physiological and pathological lymphangiogenesis [36] as well as embryonic angiogenesis before the emergence of lymphatic vessels [37]. In breast cancer, VEGFR-3 expression is upregulated in the endothelium of tumor blood vessels, while VEGF-C is highly expressed in intraductal and invasive cancer cells [38]. Notch induces VEGFR-3 expression in human endothelial cells and in mice, increasing endothelial cell responsiveness to VEGF-C and promoting endothelial cell survival and morphological changes [39]. Notch1 and Notch4 are expressed in normal and tumor lymphatic endothelial cells, and Notch1 is activated in lymphatic endothelium of invasive mammary micropapillary carcinomas [39] These data suggest a role for cross-talk between VEGFR-3 and Notch in both tumor angiogenesis and lymphangiogenesis.

### Regulation of bone marrow endothelial progenitor cells

New blood vessels formation in tumors is thought to happen through two processes: angiogenesis, defined as the proliferation and sprouting of existing blood vessels, and vasculogenesis, resulting from the recruitment of circulating cells derived from the bone marrow [40]. Endothelial progenitor cells (EPC) are an important fraction of bone-marrow derived cells in addition to myeloid cells, lymphocytes, and mesenchymal cells. Studies conducted in Jagged1-null mice have demonstrated that Jagged1 activation of Notch signaling is required for EPC development [41]. Compared to wild-type animals, Jagged1 null mice show a lower number of endothelium-specific markers expressing cells and EPC colony-forming cells [41]. Specific inactivation of Jagged1-mediated Notch signals led to inhibition of postnatal vasculogenesis in hind-limb ischemia via impairment of proliferation, survival, differentiation, and mobilization of bone marrow-derived EPCs. Recovery of hind-limb perfusion was enhanced after transplantation of Jagged1-stimulated EPCs [41]. One of the mechanisms by which activation of Notch signaling enhances mobilization and homing of EPC to neovascularization sites may be the regulation of CXCR4 expression. CXCR4 is the receptor for stromal derived factor 1 (SDF-1), a cytokine induced by hypoxia and involved in EPC homing [42]. CSL (RBP-Jκ)-deficient EPC from knockout mice have decreased ability to adhere, migrate, and form vessel-like structures in three-dimensional cultures. Over-expression of CXCR4 can rescue these defects [43]. Further evidence showing the critical role played by Notch signaling in endothelial cell maturation comes from experiments with cholesterol-lowering statins. These drugs, as a result of a pleiotropic effect, promote endothelial differentiation in bone marrow stem cells (BMSC) [44]. Simvastatin promotes the expression of endothelial markers and endothelial differentiation in BMSC. This effect can be prevented by either a γ-secretase inhibitor (GSI) or Notch1 siRNA. These data suggest that Notch1 and Jagged1 may play an important role in EPC generation and homing to tumors.

### Regulation of endothelial cell apoptosis

TNFα, a cytokine abundant in many solid tumors, cross-talks with Notch signaling in controlling endothelial cell apoptosis. In endothelial cells, TNFα treatment downregulates Notch4 mRNA and upregulates Notch2 mRNA. These changes are associated with a decrease of Notch activity, as indicated by reduced levels of Hey2 and Hes1 mRNA [45]. TNFα-mediated Notch inhibition is associated with endothelial cells apoptosis, as shown by caspase 3 activation in endothelial cells of lung sections from rats treated with TNFα. [45]. Since overexpression of Notch2 in endothelial cells decreases the levels of survivin, a key antiapoptotic factor, it has been suggested that TNFα signaling sensitizes endothelial cells to apoptosis by activating Notch2 and thus decreasing Notch activity [46]. Conversely, constitutively active Notch4 protects endothelial cells from apoptosis by increasing the levels of Bcl-2 [47]. Pulsatile flow promotes bovine retinal endothelial cells survival through Notch1 mediated upregulation of Bcl2 and Bax mRNA levels [48]. Notch signaling is also implicated in the pro-survival action of VEGF-A on endothelial cells. GSIs block the anti-apoptotic effect of VEGF-A on endothelial cells exposed to serum deprivation [49]. Additionally, Notch1 induces VEGFR-3 expression, which responds to VEGF-C promoting endothelial cells survival [39]. Thus, in addition to modulating angiogenesis and vasculogenesis, Notch signaling may control the survival of endothelial cells in tumors.

## Endothelial cells, Notch signaling and the CSC "niche"

It is becoming widely accepted that many solid tumors contain relatively rare sub-populations of cells called cancer stem-like cells (CSC), with properties similar to those of normal tissue stem cells. While the origin of these cells is controversial, there is increasing evidence that these cells are more resistant than "bulk" cancer cells to conventional therapeutic modalities and that they may be at the origin of tumor recurrence and metastasis [50]. The Notch pathway is critical in controlling the fate of CSC from several tumors and a variety of therapeutic agents targeting Notch signaling in these cells are being developed [50]. The widest experimental support to date for a role of Notch in CSC comes from studies in breast cancer [51-55], embryonal brain tumors [56], and gliomas [57,58]. Notch paralogs (1, 3 and 4) modulate breast CSC activity, with the strongest evidence favoring Notch4 [59,60]. Inhibition of Notch4 has been shown to reduce stem cell activity [61,62]. GSIs abolish the formation of secondary mammospheres from a variety of human breast cancer cell lines as well as patient specimens [63]. GSIs in combination with trastuzumab (Herceptin) abolish recurrence of Her2/Neu positive xenografts [64]. Since GSIs alone do not decrease tumor volume in this model, while trastuzumab alone drastically decreases tumor volume but does not prevent recurrence, the curative effects of GSIs most likely results from an anti-CSC effect.

The stem-like phenotype of CSC, like the stem phenotype of normal tissue stem cells, is controlled by microenvironmental signals. Endothelial cells are a major component of the CSC microenvironment, sometimes defined as a "vascular niche". It has been suggested that endothelial cells control the homeostasis of CSC by releasing stem cell-active trophogens or by direct cellular contacts (reviewed in [2]). Evidence for a role of Notch in endothelial control of CSC has been obtained in glioblastoma multiforme (GBM). In three-dimensional explants of GBM, Notch inhibition blocks the self-renewal of GBM CSC by decreasing the number of endothelial cells [65]. Conversely, CSC can stimulate angiogenesis, at least in part by producing VEGF [66-69]. Hypoxia has been suggested to play an important role in maintaining the CSC niche [70]. Hypoxia activates Notch signalling via HIF-1α in normal embryonic stem cells [71] and lung cancer cells [72], and mediates the effects of hypoxia on cancer cell fate determination in several models [73,74]. Thus, partially effective anti-angiogenic therapy, by inducing hypoxia may actually activate Notch and preserve CSC. Another facet of the endothelium/CSC interplay is the possibility that endothelial cells may be produced from trans-differentiation of CSC, a phenomenon known as vascular mimicry. This phenomenon was originally described in melanoma [75,76] and subsequently found in several other malignancies. Recent evidence indicates that glioma CSC are capable of vascular mimicry under hypoxic conditions [77], and that a significant fraction of GBM endothelial cells are derived from the tumor rather than from normal, pre-existing capillaries. A role for Notch in modulating the cell fate decisions underlying vascular mimicry has been proposed in melanoma [75] but remains poorly understood.

**Figure 2:**
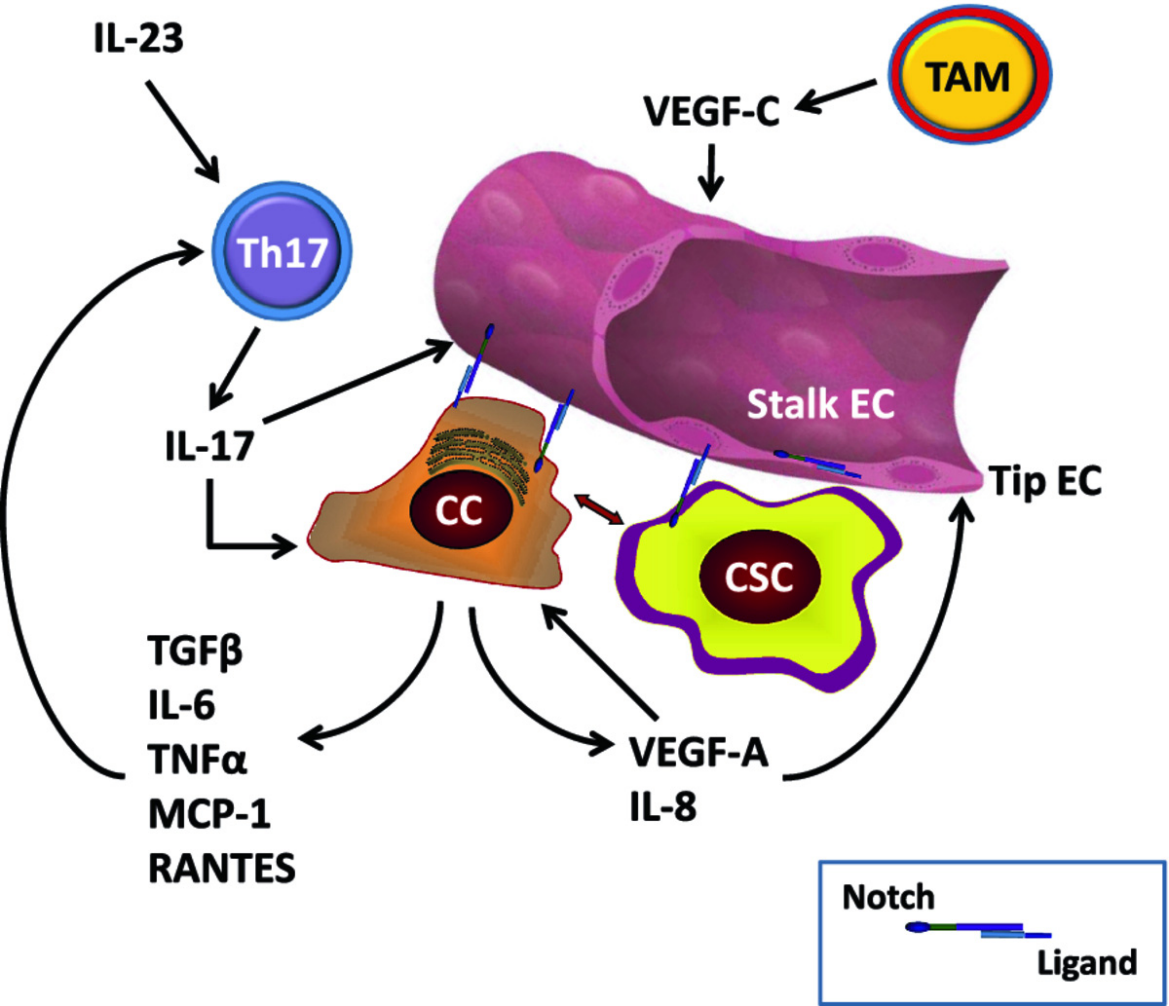
**Selected cellular interactions within the CSC niche: Endothelial cells (EC) specialize into "tip" EC, which respond to VEGF-A signals by expressing DLL4 and activating Notch in "stalk" EC, where Notch prevents further branching**. Notch-ligand interactions are represented by intercellular receptor-ligand pairs (see inset). Notch-ligand interactions can occur between tip EC and stalk EC, between CC and EC, between CSC and EC. Blood and lymphatic EC contribute to the CSC niche by providing trophic factors and ligand-Notch interactions. Non-stem cancer cells (CC) produce VEGF-A as well as numerous cytokines, including IL-8, IL-6, TNFα, MCP-1, TGF-β and RANTES. VEGF-A activates angiogenesis and has autocrine effects on cancer cells. Some cytokines (e.g., IL-8) act on EC directly, while others (e.g., IL-6, MCP-1) recruit pro-inflammatory Th17 cells. These are stimulated by IL-23 and produce IL-17, which stimulates angiogenesis. TAM produce cytokines (not shown) and VEGF-C. The latter activates VEGFR-3 in EC, stimulating Notch activity and inhibiting further branching in the context of lymphangiogenesis. Additional cells not shown in this diagram include fibroblasts, osteoclasts (in bone metastastases), bone marrow stromal cells, NK cells and others.

Cells involved in immunity and inflammation in tumor microenvironment can potentially affect both angiogenesis and CSC. Angiogenesis and immune responses are inextricably linked [78-81]. Pro-inflammatory Th17-cells, interconvertible with Th1 cells, play a crucial and complex role in tumorigenesis [79]. Tumor-infiltrating lymphocytes from human breast, ovarian and colorectal cancers contain high numbers of Th17 cells, attracted by RANTES and MCP-1 produced by tumor cells and stroma [79]. Th17 polarization requires IL-6 and IL-23, and Th17 cells produce IL-17, which stimulates angiogenesis [82,83], invasion [84] and production of pro-angiogenic IL-8 [85]. IL-6 and IL-8 have been reported to cause resistance to RO4929097 GSI [86,87]. IL-6 is a Notch target gene in tumor stroma in multiple myeloma [88] The Osborne lab in collaboration with us has shown that Notch signaling is required for the generation of Th1 [89] and Th17 [90] CD4 cells in vitro and in vivo and that GSIs inhibit Th17 lineage determination [91]. In addition to Th17 cells, other immune cell types can modulate the CSC niche, either directly or through endothelial cells. Recent evidence [92] shows that macrophage-derived VEGF-C activates VEGFR-3 in endothelial tip cells during lymphangiogenesis. VEGFR3 in turn activates Notch signaling, which promotes the phenotypic conversion of endothelial cells at fusion points of vessel sprouts. Hence, the CSC niche not only relies on endothelial cells but can itself modulate angiogenesis not only through VEGF production by cancer cells but through pro-angiogenic cytokines produced by tumor-infiltrating lymphocytes and macrophages. Tumor-associated fibroblasts also produce a variety of pro-angiogenic cytokines that modulate endothelial cell fate in the CSC niche (reviewed in [93]). The Notch pathway participates in regulating endothelial cell fate, CSC cell fate and Th17 cell fate determination, and thus plays a central role in this complex interplay. Figure 2 shows a schematic representation of these cellular interactions.

## Therapeutic implications of the cross-talk between Notch and pro-angiogenic factors in cancer

The role of Notch signaling in controlling the survival of cancer cells is well established and small molecule GSI are currently being tested in several phase 1 and 2 clinical trials in breast, lung cancer and leukemia with relatively minimal toxicity when administered intermittently [94]. We have recently concluded a pilot trial in ER+ breast cancer, where doses of GSI that did not cause significant systemic toxicity were shown to affect the expression of Notch targets and multiple CSC pathways in tumor samples [95]. Notch inhibition may block cancer growth by inhibiting the survival of both "bulk" cancer cells and CSC [5]. Cao et al. have shown that treatment with VEGF-A and GSI DAPT can re-establish responsiveness of endothelial cells to VEGF-A [96]. This implies that single-agent Notch inhibition, especially at non-cytotoxic doses, may paradoxically increase endothelial responsiveness to VEGF-A. Hypoxia, which is a likely result of VEGF inhibition, can activate Notch signaling through HIF-1α [72,74], thus potentially protecting endothelial cells from apoptosis and maintaining the integrity of existing tumor vessels, which could resume angiogenesis once VEGF inhibition is relieved. Combinations of Notch inhibitors with VEGF signaling inhibitors may provide superior anti-angiogenic activity to single-agent VEGF inhibition and deserve further study. Prolonged administration and/or high doses of GSI may be sufficient to cause endothelial apoptosis, but may be less well tolerated than lower doses or intermittent administration in combination with a VEGF inhibitor.

VEGF receptors are expressed in some human breast cancer cells and VEGF directly stimulates breast cancer progression via autocrine signaling [97,98]. We have recently reported that VEGFR-1 and -2 are expressed in a mouse ERα-positive breast cancer cell line [99,100] and that VEGF-A and VEGFRs 1and 2 are highly expressed in triple-negative breast cancer cells compared to ERα-positive breast cancer cells [100]. Additionally we confirmed that paracrine effects (especially angiogenesis) and autocrine effects (proliferation and migration) of VEGF contribute to breast cancer progression [100]. Sunitinib (SU11248), an inhibitor of protein kinases including VEGFRs 1-3, inhibits both paracrine and autocrine effects of VEGF, targeting not only the tumor vasculature but also directly inhibiting the proliferation and migration breast cancer cells *in vitro *and *in vivo *[100]. The combination of VEGF and Notch inhibitors in the treatment of breast cancer is under investigation in our lab.

## Concluding remarks and future directions

The studies presented in this review strongly suggest that angiogenic and stem cell pathways are inextricably connected in tumor microenvironment, and that the interplay between Notch and VEGF signals plays a central role in regulating cell fate within endothelial cells and CSC, as well as interactions between endothelium and CSC (Figure 2). Additionally, the role Notch-dependent pro-inflammatory Th17 cells and the role of TAM in modulating endothelial cell fate in the CSC niche requires careful investigation. Using Notch inhibitors in combination with anti angiogenic drugs in oncology could introduce a new approach to the prevention of cancer progression and recurrence by delivering synergistic anti-angiogenic effects while disrupting the CSC niche.

## Competing interests

The authors declare that they have no competing interests.

## Authors' contributions

JG reviewed the literature on tumor angiogenesis. AP reviewed the literature on cancer stem cells. PR reviewed the literature on normal endothelial and mesenchymal stem cells. BAO reviewed the literature on Th17, Th1 and tumor-associated macrophages; TEG reviewed the literature on gamma-secretase inhibitors. LM edited all author contributions, prepared the final manuscripts and drew the illustrations.
